# Primary hyperoxaluria: a case series

**DOI:** 10.1186/s13256-023-04129-z

**Published:** 2023-10-07

**Authors:** Jawad Iqbal Rather, Rabiya Rasheed, Muzafar Maqsood Wani, Mohammad Ashraf Bhat, Imtiyaz Ahmad Wani

**Affiliations:** 1 Sher-I-Kashmir Institute of Medical Sciences, Srinagar, J&k 190011 India; 2 Department of Pathology, Government Medical College, Srinagar, India

**Keywords:** Primary hyperoxaluria, Oxalosis, Nephrocalcinosis, Nephrolithiasis, Licorice, AGXT gene

## Abstract

**Background:**

Primary hyperoxaluria (PH) is a rare genetic disorder characterized by the excessive production and accumulation of oxalate. We present five cases of PH, each exhibiting varying manifestations of the disorder including a case presenting as postpartum kidney failure. Notably, three of these cases involve a previously unreported mutation.

**Case presentations:**

We evaluated five Indian patients who presented with varying manifestations of PH. The first case, a 30 year old woman, presented as post-partum kidney failure and was found to be having oxalate nephropathy precipitated by dietary oxalate overload in the setting of previously undiagnosed PH. Genetic analysis revealed a previously unreported mutation in the alanine-glyoxylate aminotransferase gene. The patient underwent simultaneous kidney liver transplant. The second and third cases, 26 and 28 year old women respectively, were asymptomatic siblings of the first patient, who were diagnosed through screening. The fourth case is a 12 year boy with PH type 1 presenting as nephrolithiasis and rapidly worsening kidney function requiring combined kidney liver kidney transplant. Case 5 is a 6 year old male child with type 2 PH presenting with nephrolithiasis, nephrocalcinosis and normal kidney function. All the patients were born to consanguineous parents.

**Conclusions:**

Due to limited clinical suspicion and inadequate diagnostic resources in certain countries with limited resources, it is possible for PH to go undiagnosed. The manifestations of the disease can range from no noticeable symptoms to severe disease. Interestingly, in some individuals with primary hyperoxaluria, the disease may not exhibit any symptoms until it is triggered by a high intake of dietary oxalate.

## Introduction

Primary hyperoxaluria (PH) is a congenital disorder characterized by a malfunction in the metabolism of glyoxylate, resulting in an augmented production of oxalate within the body. This condition leads to the excessive excretion of oxalate in urine and the accumulation of calcium oxalate in various organs. Three distinct hereditary enzymatic deficiencies have been associated with PH, namely, PH type 1 (PH1), type 2 (PH2), and type 3 (PH3) [1].

Primary hyperoxaluria type 1 (PH1) is an autosomal recessive disorder characterized by the deficiency of the hepatic enzyme alanine-glyoxylate aminotransferase. This leads to the excessive production of oxalate within the body, causing renal failure, systemic oxalate deposition, and kidney dysfunction. Among the three forms of primary hyperoxaluria, PH1 is both the most common and the most severe. It has an estimated prevalence of 1 to 3 cases per 1 million population and an incidence rate of approximately 1 case per 120,000 live births per year in Europe [2, 3]. Primary hyperoxaluria type 1 (PH1) is the most frequent and severe form of the disorder, resulting from a deficiency of the hepatic peroxisomal enzyme alanine-glyoxylate aminotransferase (AGXT). PH2 is characterized by a deficiency of glyoxylate reductase/hydroxypyruvate reductase (GR/HPR), which is encoded by the GRHPR gene. PH3, on the other hand, is caused by a deficiency of 4-hydroxy-2-oxoglutarate aldolase (HOGA), which is encoded by the HOGA1 gene [4]. Patients with PH commonly experience inflammation caused by tubular toxicity induced by oxalate. This inflammation, along with nephrocalcinosis and renal obstruction due to the formation of stones, is believed to cause the progression of chronic kidney disease (CKD). This progression sets off a vicious circle, as the decline in glomerular filtration rate (GFR) results in reduced excretion of oxalate [5]. Patients diagnosed with PH1 often develop end-stage renal disease (ESRD) at a median age of 24 years. It is worth noting that a significant proportion (20–50%) of individuals with PH1 already have ESRD at the time of their diagnosis. Moreover, the prognosis for ESRD in patients with PH1 is worse compared to those without PH1, with a roughly three-fold higher risk of mortality [6].

The standard treatment approach for PH involves a combination of medical management, dialysis, and, in ESRD, the possibility of liver and kidney transplantation [7]. However, there have been promising advancements in the field through clinical trials of novel drugs. Lumasiran, an RNA (ribonucleic acid) interference therapeutic agent, has shown positive results in Phase III clinical trials, proving its efficacy and tolerability. Consequently, it has obtained approval from both the FDA and the European Medicines Agency (EMA) for the treatment of PH1 [8].

## Case 1

A 30-year-old Indian lady, born to consanguineous parents, who had delivered a full-term baby via lower segment Caesarean Section (LSCS), presented to our department on the 4th day post-delivery with decreased urinary output, and vomiting. The pregnancy history was unremarkable, with normal blood pressure and creatinine (0.5 mg/dl in the third trimester). There was no history of fever or postpartum hemorrhage. After delivery, the patient reported consumption of an herbal concoction that included licorice and some other herbs. On examination, she had tachycardia, tachypnea, a blood pressure of 140/90 mmHg, and 2+ pitting edema in her legs. Chest examination revealed bilateral basal crepts. On investigation, she had a hemoglobin of 9.1 g/dl with MCV 87 fl and MCHC 31 g/dl. Her total leukocyte count was 6300/microlitre, and her platelet count was 170,000/microlitre. Her urea was 190 mg/dl and her creatinine was 15.5 mg/dl. Total bilirubin was 0.8 mg/dl. AST (aspartate aminotransferase) and ALT (alanine aminotransferase) were 17 IU/L and 13 IU/L, respectively. Her direct Coomb’s test was negative. The blood gas analysis revealed metabolic acidosis. Urine examination revealed proteinuria 1+, 2–3 red cells/hpf (high power field), leukocytes 10–12/hpf, granular casts, and oxalate crystals. Ultrasound imaging showed normal-sized kidneys with a raised echo-pattern. There was not any hydronephrosis, and renal arterial and venous Doppler was normal. The spot urine protein creatinine ratio was 0.6. The peripheral blood film was negative for schistocytes. Antinulclear antigen (ANA), anti-myeloperoxidase (MPO), anti-proteinase 3 (PR3), and anti-glomerular basement membrane (GBM) antibodies were negative. Complement levels were within the normal range.

**Fig. 1:**
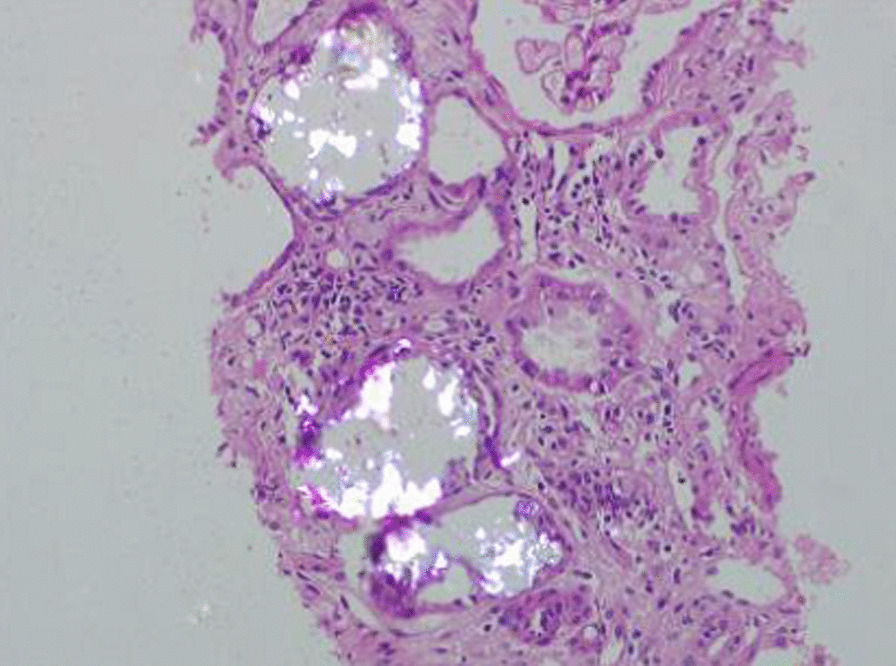
Extensive refractile oxalate crystals is noted in tubular lumina

Hemodialysis was initiated, and an ultrasound-guided percutaneous kidney biopsy was done. Light microcopy revealed 7 glomeruli out of which 3 were globally sclerosed. Significant proliferative or exudative activity was not seen in viable glomeruli. Tubules showed prominent cytoplasmic vacuolar change and severe acute injury with epithelial simplification and loss of brush borders. Extensive inspissation of refractile oxalate crystals was noted in tubular lumina and cytoplasm. Interstitial fibrosis and atrophy involved about 40% of sampled cortex (Fig. 1). Immunofluorescence study was negative. 24-h urinary oxalate excretion was 210 mg (reference value 7.0–44.0 mg) by spectrophotometry. The serum osmolal gap was within normal limits.

Due to the elevated levels of oxalate and consanguinity, a clinical exome study was performed, which identified a homozygous duplication of 14 base pairs in exon 9 of the AGXT gene (c.869_882 duplication). This duplication causes a frame-shift mutation and premature truncation of the protein, specifically 22 amino acids downstream of codon 295 (p.Ala295SerfsTer22). Based on these findings, a diagnosis of primary hyperoxaluria type 1 (PH1) was confirmed. The patient was initially managed with intensive hemodialysis and subsequently underwent a combined liver and kidney transplant at a different centre.

## Case Nos. 2 and 3

The siblings (aged 26 years and 28 years Indian, respectively) of case no. 1 were investigated, which revealed 24-h urinary oxalate excretion of 110 mg, and 95 mg, respectively (reference value 7.0–44.0 mg) by spectrophotometry. Serum creatinine was 0.57 mg/dl and 0.73 mg/dl, corresponding to estimated glomerular function rate (eGFR) of 128 ml/min/1.73 m^2^ and 115 ml/min/1.73 m^2^, respectively. On imaging, no nephrolithiasis was found in either of the siblings. The same mutation was found as in case 1.

They were counseled about the diagnosis with the advice of hyperhydration, potassium citrate, balanced diet and pyridoxine supplementation. Both cases were lost to follow-up.

## Case No. 4

A 12-year-old Indian, born to consanguineous parents with no significant perinatal history, normal developmental milestones for age, and no significant family history, presented with vomiting and azotemia. His laboratory tests were: urea 62 mg/dl, creatinine 1.8 mg/dl (eGFR 31 ml/min/1.73 m^2^), corrected calcium 8.9 mg/dl, phosphorus 4.25 mg/dl, Na 139 mEq/L, potassium 4.9 mEq/L, and bicarbonate 17 mmol/L. His haemoglobin was 12 g/dl. Urine examination revealed proteinuria (traces), 6–8 RBCs/hpf, and leukocytes (18–20/hpf). Ultrasonography revealed increased echogenicity and bilateral renal and ureteric calculi. His parathyroid hormone (PTH) level was 71 pg/ml (reference value 14–72 pg/ml); vitamin D levels were normal; and his 24-h urinary oxalate level was high (180 mg/day), which was confirmed twice. 24-h levels for calcium, citrate, phosphorus, and uric acid were within the normal range. Ureteroscopic lithotripsy was done, and stone analysis revealed a composition of 90% calcium oxalate monohydrate and 10% calcium oxalate dihydrate. However, his creatinine did not decrease, and it progressively worsened post-ureteroscopy. He was subsequently started on hemodialysis.

Considering the elevated levels of urinary oxalate and the presence of consanguinity, a genetic analysis was performed, which identified a homozygous missense variant in exon 2 of the AGXT gene (c.302T.C). This variant causes an amino acid substitution, specifically replacing leucine with proline (p.Leu101Pro). He did not respond to pyridoxine. Based on these findings, the patient underwent a combined liver and kidney transplant.

## Case No. 5

We received a 6-year-old Indian male patient who was born to consanguineous parents. The child presented to our outpatient department with complaints of bilateral flank pain. Ultrasound revealed bilateral nephrolithiasis and nephrocalcinosis. His creatinine was 0.67 mg/dl, corresponding to eGFR (68 ml/min/1.73 m^2^). His PTH was 56 pg/ml, and his vitamin D was 35 ng/ml. Urine examination revealed 8–10 leukocytes and 6–7 red blood cells per high-power field. He underwent ureteroscopic lithotripsy, and stone analysis revealed a 90% and 10% calcium oxalate monohydrate and calcium oxalate dihydrate composition, respectively. His 24-h urinary calcium, phosphate, citrate, and urate levels were normal. The 24-h urinary oxalate level was 124 mg.

Because of high oxalate levels and consanguinity, genetic analysis was done, which revealed a homozygous missense splice-site proximal variation in exon 6 of the GRHPR gene (c.494G > A) that results in the amino acid substitution of aspartic acid for glycine at codon 165 (p. Gly165Asp).

## Discussion

PH1 is characterized by a deficiency of the hepatic-specific peroxisomal enzyme alanine-glyoxylate aminotransferase (AGXT). AGXT is an enzyme that depends on pyridoxal 5'-phosphate and plays a crucial role in converting glyoxylate to glycine through transamination. In PH1, due to the reduced transamination activity of glyoxylate to glycine, there is an accumulation of glyoxylate, leading to increased production of oxalate [9].

In India and other developing countries, primary hyperoxaluria (PH) continues to pose a diagnostic challenge. The incidence and prevalence of the condition remain unknown, primarily due to its wide spectrum of clinical presentations and the ability to manifest at any age. As observed in the five cases mentioned above, PH can present with various clinical manifestations. These can range from severe manifestations such as infantile massive nephrocalcinosis and failure to thrive, to milder presentations with recurrent or occasional stone formation. Additionally, some individuals may remain asymptomatic, while others may progress to end-stage renal disease (ESRD) as their first noticeable symptom [2, 10, 11]. Recurrent urolithiasis or progressive nephrocalcinosis is usually one of the first symptoms and, if supported by a suggestive family history or consanguinity, should raise possibility of PH1 and prompt one to go for further screening [2, 11].

As seen with various studies, a mean of 5 years is observed from the onset to the final diagnosis [9], a finding consistent with the cases in our series, and we attribute that to clinical heterogeneity, a lack of clinical suspicion, and the rarity of the disease [11]. An early diagnosis remains vital to preventing or slowing down the end-stage disease.

Case No. 1, discussed above, gave a history of the intake of large quantities of some local herbal concoction over a period of 2 days, which among many other things, contained licorice. Licorice extract is often used in traditional medicine to treat conditions like inflammation, gastric ulcers, and respiratory diseases [10]. It is well known that excessive consumption of licorice can cause hypertension and hypokalemia [12]. Licorice root is also one of the highest oxalate-containing foods. (3569.3 mg/100 g) [13]. Before the consumption of licorice, the case belonged to the pre-symptomatic group of PH1, which constitutes about 13% of all cases of PH1 [14]. The patient did not show any stones on kidney imaging. A kidney biopsy showed extensive inspissations of refractile oxalate crystals in tubular lumina and cytoplasm (Fig. 1). Acute intake of large doses of an oxalate-rich concoction over the previous 2 days compared to her regular diet explained the worsening of kidney function. The patient underwent a kidney biopsy, which revealed oxalate nephropathy. To further confirm the diagnosis, genetic testing was conducted and revealed a homozygous duplication of 14 base pairs in exon 9 of the AGXT gene. This duplication leads to a frameshift mutation and premature truncation of the protein, occurring 22 amino acids downstream of codon 295. These findings provide genetic evidence supporting the diagnosis of oxalate nephropathy in the patient. The AGT enzyme is encoded by a single copy gene called AGXT, which consists of 11 exons. These exons range in size from 65 base pairs to 407 base pairs and span over a 10-kilobase DNA segment in the 2q37.3 region of the human genome [15]. To date, more than 190 clinically significant mutations have been identified in the AGXT gene. Among these mutations, three of the most common worldwide are p.G170R, c.33dupC, and p.I244T. These mutations account for approximately 30%, 11%, and 6% of AGXT mutant alleles, respectively. These observations suggest the existence of mutation "hot spots" in exons 4, 1, and 7 of the AGXT gene [16, 17]. However, our patient had a mutation in exon 9 of the AGXT gene, with the variant reported as AGXT p.Ala295SerfsTer22 (homozygous, nonsense), which has not been reported earlier in the literature or Clinvar database. But other truncating variants in the AGXT gene are reported as pathogenic in the Clinvar database for primary hyperoxaluria. As loss of function mutations are known to cause this condition, this variant was classified as a pathogenic variant. The patient underwent a combined liver and kidney transplant.

The clinical exome study conducted on Cases No. 2 and 3, who are siblings of the first case, yielded the same mutation findings. Both individuals were found to have a homozygous 14 base pair duplication in exon 9 of the AGXT gene. Interestingly, despite carrying the mutation, both siblings were asymptomatic. This highlights the wide clinical spectrum of primary hyperoxaluria type 1 (PH1), ranging from being asymptomatic with occasional stone formation to severe presentations [18].

True to its phenotypic and genotypic heterogeneity, in our case 4, a young 12-year-old boy presented with a history of recurrent bilateral renal calculi. Screening for primary hyperoxaluria (PH) is rarely performed in Indian children, despite the occurrence of nephrocalcinosis and nephrolithiasis [19, 20]. The genetic analysis revealed a homozygous missense variant in exon 2 of the AGXT gene (c.302T.C), leading to the substitution of leucine with proline at codon 101. Previous reports in the literature have also described North Indian children with primary hyperoxaluria Type 1 who were homozygous for the c.302T > C (p.Leu101Pro) AGXT mutation [20, 21].

PH2 is caused by a deficiency of the enzyme glyoxylate reductase/hydroxypyruvate reductase (GR/HPR) [22–24]. GR/HPR enzyme activity is highest in the liver but also is demonstrated in other tissues [25]. It is primarily located in the cytosol but is also found in the mitochondria [26]. Increased urine l-glycerate excretion is characteristic for this disorder, although false-negative results have been described [27]. Sometimes patients with hyperoxaluria are misclassified as having PH1 solely based on disease severity but one must differentiate it from PH2 [28]. The reported median ages of first-noticed symptoms in PH2 vary from 3.2 to 7.4 years. The age of our case is 6 years. The manifestations range from kidney stones and nephrocalcinosis to systemic oxalosis, with kidney stones and nephrocalcinosis occurring in more than 80% of cases of PH2. About one-quarter to one-third of PH2 patients progress to ESKD.

## Conclusions

1. Because of low clinical suspicion and lack of diagnostics in resource-limited countries, PH may be missed as a diagnosis.
2. The disease presentation may vary from being asymptomatic to massive nephrocalcinosis, nephrolithiasis, and ESKD as the first presentation.
3. In some patients with primary hyperoxaluria, the disease may be asymptomatic and can be precipitated by a high dietary oxalate load as seen in one of our patients.

## Data Availability

Data regarding genetic mutations is available.

## References

[1] Harambat J, Fargue S, Bacchetta J, Acquaviva C (2011). Primary hyperoxaluria. Int J Nephrol..

[2] Cochat P, Deloraine A, Rotily M, Olive F (1995). Epidemiology of primary hyperoxaluria type 1. Nephrol Dial Transplant..

[3] van Woerden CS, Groothoff JW, Wanders RJ (2003). Primary hyperoxaluria type 1 in The Netherlands: prevalence and outcome. Nephrol Dial Transplant.

[4] Lorenzo V, Torres A, Salido E (2014). Primary hyperoxaluria. Nefrologia..

[5] Mulay SR, Kulkarni OP, Rupanagudi KV, Migliorini A (2013). Calcium oxalate crystals induce renal inflammation by NLRP3-mediated IL-1β secretion. J Clin Invest.

[6] Harambat J, van Stralen KJ, Espinosa L (2012). Characteristics and outcomes of children with primary oxalosis requiring renal replacement therapy. Clin J Am Soc Nephrol.

[7] https://www.biospace.com/article/releases/alnylam-receives-approval-for-oxlumo lumasiran-in-the-european-union-for-the-treatment-of-primary-hyperoxaluria-type-1-in-all-age-groups/

[8] Zhang X, Roe SM, Pearl LH (2001). Crystallization and preliminary crystallographic analysis of human alanine:glyoxylate aminotransferase and its polymorphic variants. Acta Crystallogr D Biol Crystallogr.

[9] Soliman NA, Nabhan MM, Abdelrahman SM (2017). Clinical spectrum of primary hyperoxaluria type 1: experience of a tertiary center. Nephrol Ther.

[10] Kurt-Sukur ED, Özçakar ZB, Fitöz S (2015). Primary hyperoxaluria type 1: a cause for infantile renal failure and massive nephrocalcinosis. Klin Padiatr.

[11] Yang R, Wang LQ, Yuan BC (2015). The pharmacological activities of licorice. Planta Med.

[12] Sontia B, Mooney J, Gaudet L (2008). Pseudohyperaldosteronism, liquorice, and hypertension. J Clin Hypertens (Greenwich).

[13] Siener R, Seidler A, Hönow R (2021). Oxalate-rich foods. Food. Sci Technol.

[14] Ganhão I, Borges C, Amorim M (2020). Primary hyperoxaluria type 1 – two case reports. Port J Nephrol Hypert..

[15] Lu-Kuo J, Ward DC, Spritz RA (1993). Fluorescence in situ hybridization mapping of 25 markers on distal human chromosome 2q surrounding the human Waardenburg syndrome, type I (WS1) locus (PAX3 gene). Genomics.

[16] Hopp K, Cogal AG, Bergstralh EJ, Seide BM, Rare Kidney Stone Consortium (2015). Phenotype-genotype correlations and estimated carrier frequencies of primary hyperoxaluria. J Am Soc Nephrol..

[17] Nagara M, Tiar A, Ben Halim N, Ben Rhouma F, Messaoud O, Bouyacoub Y, Kefi R, Hassayoun S, Zouari N, Ben Ammar MS, Abdelhak S, Chemli J (2013). Mutation spectrum of primary hyperoxaluria type 1 in Tunisia: implication for diagnosis in North Africa. Gene.

[18] Alfadhel M, Alhasan KA, Alotaibi M (2012). Extreme intrafamilial variability of Saudi brothers with primary hyperoxaluria type 1. Ther Clin Risk Manag.

[19] Mantan M, Bagga A, Virdi VS (2007). Etiology of nephrocalcinosis in northern Indian children. Pediatr Nephrol.

[20] Chanchlani R, Sinha A, Gulati A (2012). Common mutation underlying primary hyperoxaluria type1 in three Indian children. Indian J Nephrol.

[21] Sethi SK, Waterham HR, Sharma S (2009). Primary hyperoxaluria type 1 with a novel mutation. Indian J Pediatr.

[22] Williams HE, Smith LH (1968). L-glyceric aciduria. A new genetic variant of primary hyperoxaluria. N Engl J Med..

[23] Mistry J, Danpure CJ, Chalmers RA (1988). Hepatic D-glycerate dehydrogenase and glyoxylate reductase deficiency in primary hyperoxaluria type 2. BiochemSoc Trans.

[24] Cramer SD, Ferree PM, Lin K (1999). The gene encoding hydroxypyruvate reductase (GRHPR) is mutated in patients with primary hyperoxaluria type II. Hum Mol Genet.

[25] Giafi CF, Rumsby G (1998). Kinetic analysis and tissue distribution of human D-glycerate dehydrogenase/glyoxylate reductase and its relevance to the diagnosis of primary hyperoxaluria type 2. Ann Clin Biochem.

[26] Behnam JT, Williams EL, Brink S (2006). Reconstruction of human hepatocyte glyoxylate metabolic pathways in stably transformed Chinese-hamster ovary cells. Biochem J.

[27] Rumsby G, Sharma A, Cregeen DP (2001). Primary hyperoxaluria type 2 without L-glycericaciduria: is the disease under-diagnosed?. Nephrol Dial Transplant.

[28] Milliner DS, Wilson DM, Smith LH (2001). Phenotypic expression of primary hyperoxaluria: comparative features of types I and II. Kidney Int.

